# The evaluation of artificial intelligence in mammography-based breast cancer screening: Is breast-level analysis enough?

**DOI:** 10.1007/s00330-025-11733-8

**Published:** 2025-06-25

**Authors:** Adnan Gani Taib, George John William Partridge, Luyan Yao, Iain Darker, Yan Chen

**Affiliations:** Translational Medical Sciences, School of Medicine, University of Nottingham, Clinical Sciences Building, City Hospital Campus, Hucknall Road, Nottingham, NG5 1PB UK

**Keywords:** Artificial intelligence, Mammography, Breast neoplasms, Mass screening

## Abstract

**Objectives:**

To assess whether the diagnostic performance of a commercial artificial intelligence (AI) algorithm for mammography differs between breast-level and lesion-level interpretations and to compare performance to a large population of specialised human readers.

**Materials and methods:**

We retrospectively analysed 1200 mammograms from the NHS breast cancer screening programme using a commercial AI algorithm and assessments from 1258 trained human readers from the Personal Performance in Mammographic Screening (PERFORMS) external quality assurance programme. For breasts containing pathologically confirmed malignancies, a breast and lesion-level analysis was performed. The latter considered the locations of marked regions of interest for AI and humans. The highest score per lesion was recorded. For non-malignant breasts, a breast-level analysis recorded the highest score per breast. Area under the curve (AUC), sensitivity and specificity were calculated at the developer’s recommended threshold for recall. The study was designed to detect a medium-sized effect (odds ratio 3.5 or 0.29) for sensitivity.

**Results:**

The test set contained 882 non-malignant (73%) and 318 malignant breasts (27%), with 328 cancer lesions. The AI AUC was 0.942 at breast level and 0.929 at lesion level (difference −0.013, *p* < 0.01). The mean human AUC was 0.878 at breast level and 0.851 at lesion level (difference −0.027, *p* < 0.01). AI outperformed human readers at the breast and lesion level (*ps* < 0.01, respectively) according to the AUC.

**Conclusion:**

AI’s diagnostic performance significantly decreased at the lesion level, indicating reduced accuracy in localising malignancies. However, its overall performance exceeded that of human readers.

**Key Points:**

***Question*** *AI often recalls mammography cases not recalled by humans; to understand why, we as humans must consider the regions of interest it has marked as cancerous.*

***Findings*** *Evaluations of AI typically occur at the breast level, but performance decreases when AI is evaluated on a lesion level. This also occurs for humans.*

***Clinical relevance*** *To improve human-AI collaboration, AI should be assessed at the lesion level; poor accuracy here may lead to automation bias and unnecessary patient procedures.*

**Graphical Abstract:**

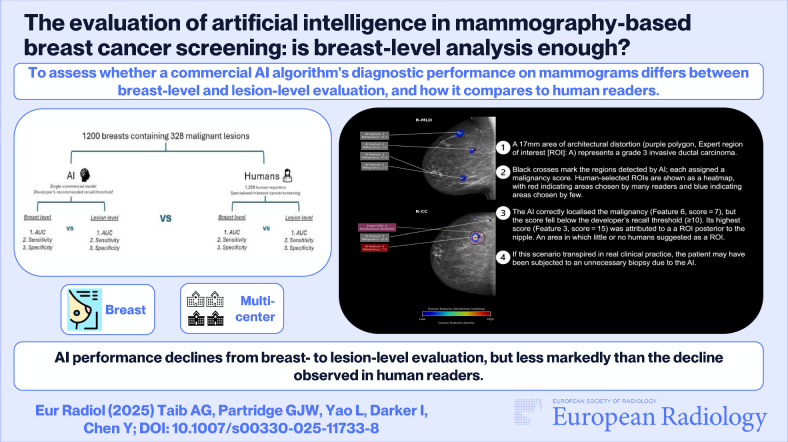

## Introduction

Pooled retrospective studies have confirmed that the diagnostic capability of artificial intelligence (AI) in mammography-based breast cancer screening is superior or comparable to humans [1]. This suggests some AI models may be useful for clinical practice. As such, AI is now being integrated into real-life screening workflows prospectively [2–4]. The next critical step is to explore its working relationship with humans and how AI may influence the decision-making process. However, concern still exists surrounding its lack of explainability, commonly referred to as the “black box” phenomenon [5]. One method that may alleviate this is to ensure AI displays its regions of interest (ROI) on a mammogram.

Most studies evaluate AI and human performance at breast level during mammography, focussing only on an image-level classification i.e. the presence of cancer or not [6]. Very few studies consider the accuracy of ROIs marked by AI and humans on the mammogram [2, 7, 8], known as a pixel-level classification [6]. During real-life screening, a human reporter assigns an image level score for a breast but is also asked to localise the ROI. This enables those involved in the arbitration or follow-up of a case to understand the original reader’s “working out.” Likewise, if AI is to be implemented into a screening workflow, an element of explainability is key in understanding incongruent cases, particularly where AI has suggested a ROI, but a human has not [3]. Therefore, AI should be assessed in its ability to accurately localise lesions, enabling comparison with the location-specific ratings provided by humans [6].

The aim of this study is to assess whether the diagnostic performance of a commercial AI algorithm differs in its interpretation of mammograms for breast cancer when evaluated at breast-level and lesion-level using the same dataset. Additionally, we compared AI’s performance to a large sample of specialised human readers. Human performance was evaluated within the Personal Performance in Mammographic Screening (PERFORMS) external quality assurance scheme (EQA) [9].

## Methods

### Study sample

The research was conducted adhering to the local information system security and data protection policies. Funding for this research article was provided by Lunit, which had no involvement in its production.

Ten consecutive PERFORMS test sets (i.e. schemes), each consisting of mammograms from 60 patients, were interpreted by human readers in the UK as part of mandatory and routine quality assurance for the National Health Service Breast Screening Programme (NHSBSP) between 2012 and 2023. The AI read the same 600 cases in 2023 as an independent reader.

Any mammography reader in the UK NHSBSP who participated in a single PERFORMS scheme between 2012 and 2023 was considered for inclusion in the study. The NHSBSP routinely invites women between the ages of 50 and 70 years to undergo two-view two-dimensional digital mammography every 3 years. Radiologists and radiographers account for 94% of mammogram readers in the NHSBSP [10]. Radiographers interpreting screening mammograms undergo advanced training, typically to a masters’ level and work as consultant radiographers and advanced practitioners. Breast clinicians are medically qualified physicians who are not radiologists but are specialised in diagnosing and treating breast disease [11]. Therefore, readers included in this study consisted of board-certified radiologists, specialty-trained radiographers and breast clinicians. In the National Health Service Breast Screening Programme (NHSBSP), it is mandated that reporters read a minimum of 5000 mammograms per year [12]. Non-board-certified radiologists and those involved in symptomatic breast reporting only were excluded. Each scheme was read by a different number of humans. However, the same human may have undertaken multiple schemes. Each human was, therefore, classed as an independent entity per the scheme.

### PERFORMS test set images

The PERFORMS test sets are derived from an anonymised pool of mammograms contributed by UK screening centres across the NHSBSP. Each scheme consisted of 60 challenging mammogram patient cases enriched with cancers. Organisers of PERFORMS, alongside a panel of experienced breast radiologists (with over 20 years’ screening experience), selected the mammograms for each scheme, drew the ROIs based on pathology reports of the biopsy locations and determined the breast density for each case [13]. An expert radiologist with over 20 years’ experience more recently re-reviewed all malignant lesions (*n* = 328) against their biopsy locations and revalidated the ROI hitboxes to ensure veracity of the metadata in 2023.

The selected full field digital mammograms (FFDMs) in the PERFORMS schemes are uploaded to each screening centre’s picture archiving and communication system for interpretation by human readers in their clinical environment. Findings, including abnormality locations, are recorded on a secure, password-protected website [13].

### Human scoring system

Humans provided scores for each breast and ROI independently on a scale of 1 to 5 as per the Royal College of Radiologists (RCR) classification system: 1 normal, 2 benign, 3 indeterminate, 4 suspicious and 5 malignant. A score of 1 or 2 indicated that the reader considers the case normal or benign, meaning the case should return to screening. If a score of 3, 4 or 5 was assigned, the reader thought the case should be recalled for further investigation [14]. The PERFORMS platform allows participants to assign an RCR rating per breast and per lesion. The platform automatically updates the breast level rating to the highest rated ROI assigned to a lesion. In circumstances where the ROI is scored with a lower RCR rating than the breast, a mismatch may occur. Therefore, a lesion level reading for a human was excluded if the human marked a feature in the ROI with a score of 1, but the RCR score for the breast was > 1. If a human had > 15% of their lesion level readings excluded for a scheme, their entire scheme read was excluded to ensure the veracity of lesion level sensitivity across all humans.

### AI scoring system

The AI model used in this study was Lunit Insight MMG V1.1.7.1. The AI had no access to the cases during its training or development, either before or after the study. AI acted as an independent reader of cases. Suspicion of malignancy scores ranging from 0 (lowest suspicion) to 100 (highest suspicion) were assigned to each lesion detected by the AI. The threshold for recall was set at a developer’s recommended score of ≥ 10 [15]. Other recall thresholds for AI were tested to match average human reader sensitivity(≥ 10.5) and specificity (≥ 4.5). If a breast had no lesions of interest according to the AI, it was assigned a score of 0.

### Statistical analysis

The human and AI were compared in terms of the binary decisions to return or recall a breast or lesion for further investigation against the ground truth for a case: malignant or not, based on case pathology or a normal follow-up at 3 years. The α level a priori power of the analysis to detect a medium-sized effect (odds ratio (OR), 3.5 or 0.29) in terms of cancers missed (i.e., 1—sensitivity) by AI and the average human reader was 99%, based on the average human reader missing 10% of cancers.

### Area under the receiver operating characteristic curve (AUC) analyses

AUC was calculated at the breast and lesion levels for AI and humans using the trapezoid method for each of the ten schemes independently. These were then compared using a *z*-test score for distribution, with its related *p*-value taken from the normal distribution. For humans, Stouffer’s method was applied to combine *z*-scores from the ten independent schemes to assess the overall effect across multiple test sets [16, 17]. Each scheme was weighted equally, given the uniform process used for selection of FFDM cases by experts and the similar proportions of malignant lesions in each test set. Approximately one quarter of the breasts contained at least one malignant lesion.

### AI and average human reader performance analyses

Average human sensitivity was derived by calculating the mean human sensitivity of a scheme and then multiplying this proportion by the number of malignant lesions or breasts within that scheme. This provided the mean number of malignancies detected per scheme. The mean numbers of cancers detected in each scheme were then summed to provide an average “modelled” number of true positive (TP) cancer lesions detected out of all cancers in the case set. A similar method was used to calculate the mean “modelled” specificity for all non-malignant breasts using the true negative (TN) rate for each scheme independently and then combining them. The sensitivity and specificity of AI were compared to the “modelled” average human reader using an OR.

### Lesion-level analyses

An automated “hit detection” programme was developed to extract the location and scores of all ROIs marked by each human and AI on every FFDM. More information regarding this programme is available in the supplementary file.

#### Sensitivity

**Fig. 1:**
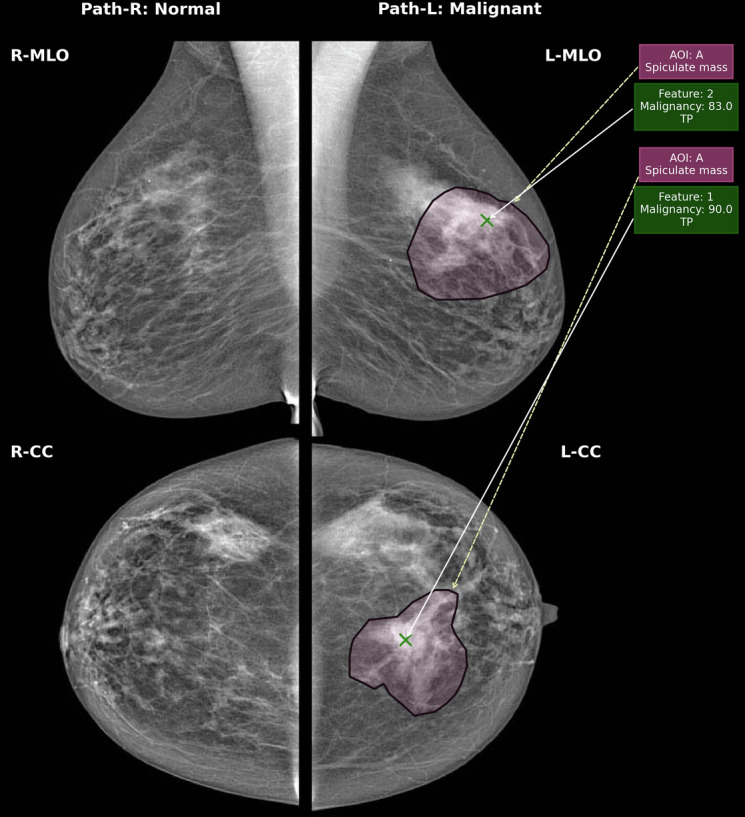
An example of the output from the hit detection programme used to extract reader information. In the example, the right breast is a normal breast, and therefore, this was analysed at breast level for specificity. In the left breast, a malignant grade 2 invasive lobular carcinoma is visualised in both views, this was analysed at lesion level for sensitivity. This considers both the location of the ROI (i.e. if it sits within or close to the pathologically confirmed ROI) and its rating. In the right breast, AI has not marked any ROI, so it would be assigned a score of 0, a TN. In the left breast, AI has assigned a score of 83 and 90 in the mediolateral oblique (MLO) and craniocaudal (CC), respectively (green crosses). As the highest score is taken per malignant lesion, a score of 90 would be assigned to this lesion by AI in this study, meaning it would be recalled as a TP

Pathologically confirmed malignant ROIs were marked by a polygonal hitbox on a FFDM. ROIs for the same lesion could be drawn on the mediolateral oblique (MLO) and craniocaudal (CC) or both views. A TP was defined as the reader placing a mark within a malignant ROI and assigning a rating greater than the threshold for recall. The highest score for a cancer lesion within a breast, in either view, was taken as the overall score for the lesion (Fig. 1). A score lower than the threshold for recall for a correctly placed marker was classified as a false negative (FN). Any malignant ROI not receiving a mark within its hitbox was assigned a human RCR rating of 1 or an AI rating of 0, also classifying it as an FN. For breasts containing malignant ROIs, the protocol did not consider AI or human marks outside of the hitbox, as this may have contained malignancies not detected by human screeners at the time. For humans and AI, lesion-wise sensitivity was calculated as the percentage correctly localised and recalled malignant lesions, over the total number of malignant lesions.

#### Specificity

In non-malignant (normal or benign) breasts, ROIs were not considered, therefore specificity was analysed at breast level (Fig. 1).

### Breast level analyses

The highest rating assigned by a reader for a breast, across both views, was taken as the overall score for that breast. Each breast was considered independently.

#### Sensitivity

If a score was assigned to a breast containing a malignant lesion above the respective recall threshold, it was classified as a TP. A score below the recall threshold for a breast containing cancer was classified as an FN. Sensitivity was classified as the percentage of correctly recalled breasts over the total number of breasts containing malignancy.

#### Specificity

If a score was assigned to a non-malignant breast below the respective thresholds for recall, it was deemed a TN. If scores exceeded the threshold, they were classified as a false positive (FP). Specificity was classified as the percentage of non-malignant breasts returned to screen correctly, out of all non-malignant breasts.

The 95% CIs and *p*-values for the ORs were calculated as described by Kirkwood and Sterne [18]. All other statistical calculations were performed using IBM SPSS Statistics (version 27.0). All statistical tests were two-sided. For all analyses, the *α* level for statistical significance was < 0.05. Statistical analysis was conducted by author A.G.T.

## Results

### Demographics

**Fig. 2:**
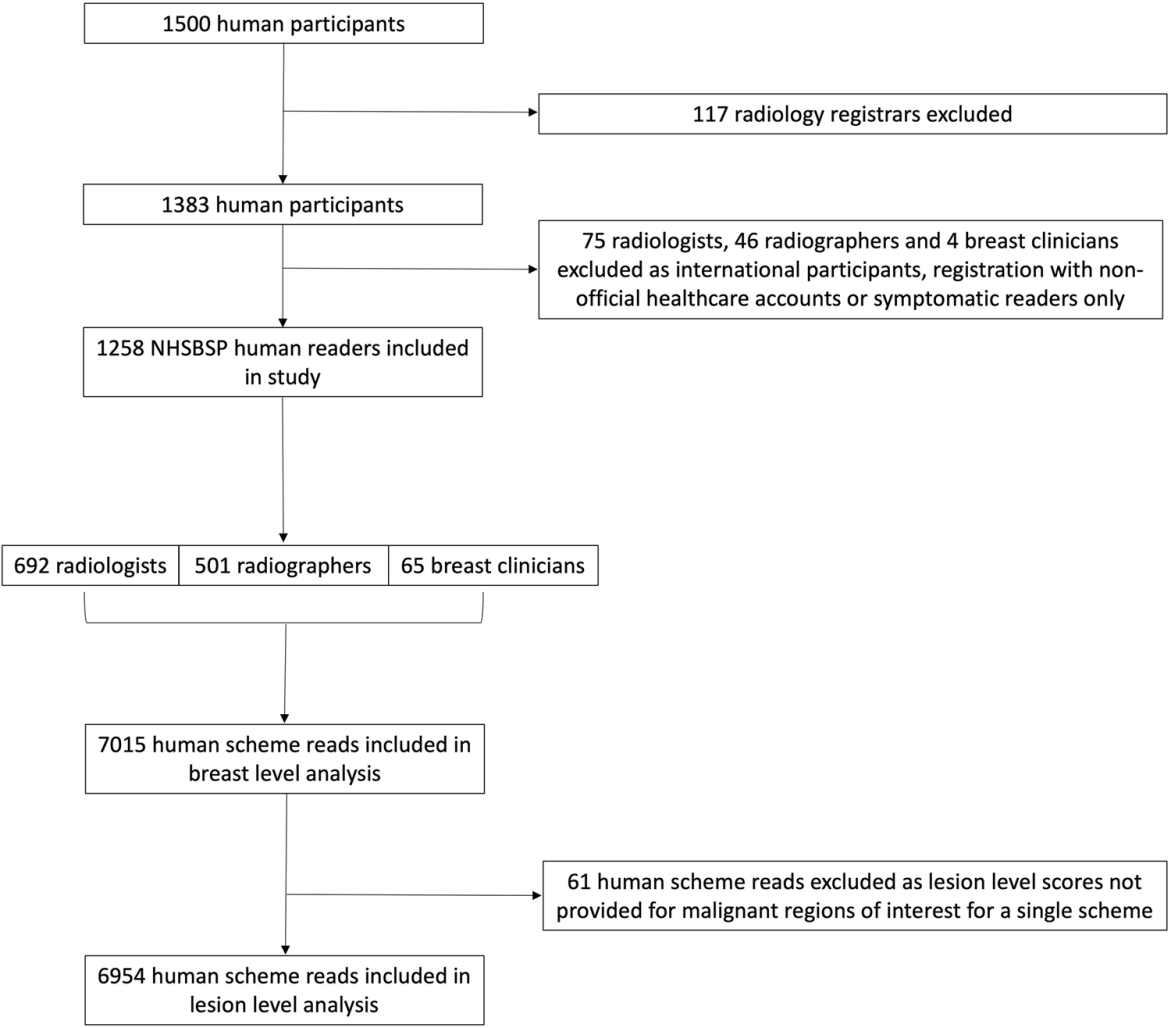
Flowchart illustrating inclusion and exclusion criteria. NHSBSP, National Health Service Breast Screening Programme; PERFORMS, Personal Performance in Mammographic Screening

One thousand five hundred human readers were eligible for inclusion in the study. Radiology registrars (*n* = 117) and PERFORMS participants not reporting in the NHSBSP (*n* = 125) were excluded from the study. Therefore, a total of 1258 UK human screening FFDM reporters were compared to the AI. This included 692 board-certified radiologists (55%), 501 radiographers (40%) and 65 breast clinicians (5%), Fig. 2. In total, there were 7015 human reads of at least one scheme included in the study. Sixty-one human scheme reads were excluded at the lesion level analysis as they did not assign an independent score to ROIs in the respective scheme, resulting in 6954 human reads at the lesion level. This included four humans excluded entirely from lesion-level analysis for not assigning ROI scores in any of their schemes.

Table 1 provides details on the pathological and radiographic features of the 600 PERFORMS cases. There were 318 malignant breasts in the test set (27%), containing a total of 328 cancer lesions. Normal (*n* = 827 (69%)) or benign (*n* = 55 (5%)) breasts were analysed at breast level. Of the malignant lesions, four-fifths were invasive (264 of 328 lesions (80%)), and ductal was the most common pathological subtype of cancer (229 of 328 lesions (70%)). The median size of the malignant lesions was 13 (interquartile range 8 mm). Masses were the predominant radiological feature of malignant lesions (194 of 328 lesions (59%)), followed by calcifications (58 of 328 lesions (18%)) and architectural distortions (57 of 328 lesions (17%)). Breasts with density category A and B accounted for 67% of the test set (808/1200).

**Table 1:** Pathological and radiological features of the mammography cases

Breast pathological outcome	Finding (*n*)	%
Normal (breast count)	827	69
Benign (breast count)	55	5
Malignant (breast count)	318	27
Malignant (lesion count)	328	-
Invasive cancer		
In situ	62	19
Invasive	264	80
Missing data	2	1
Cancer type		
Ductal	229	70
Lobular	44	13
Other	36	11
Missing data	19	6
Grade		
1	85	26
2	149	45
3	59	18
Missing data	35	11
Size of malignant lesion (median, Q1, Q3, mm)	13 (9, 17)	-
^a^Size of benign lesion (median, Q1, Q3, mm)	12 (6, 22)	-
Radiological feature of malignant lesions		
Spiculate mass	103	31
Ill-defined mass	78	24
Well-defined mass	13	4
Calcification	58	18
Architectural distortion	57	17
Asymmetry	19	6
Breast density (breast level)		
A: < 25%, almost entirely fatty	328	27
B: 25%–50%, scattered fibroglandular densities	480	40
C: 51%–75%, heterogeneously dense	302	25
D: > 75%, extremely dense	90	8
Mammography vendor (breast level)		
Fischer Imaging Corporation	20	2
Fujifilm	62	5
GE	382	32
Hologic	364	30
Phillips	52	4
Sectra	22	2
Siemens	116	10
Missing data	182	15

Categorical data are presented as proportions of breasts or malignant lesions, and continuous data are presented as median ± quartile 1, quartile 3

*PERFORMS* Personal Performance in Mammographic Screening, *mm* millimetres, *Q1* quartile 1, *Q3* quartile 3

^a^ 8 benign cases missing size measurement for dominant lesion

### Comparison of the area under the receiver operating curve at the breast and lesion level

**Fig. 3:**
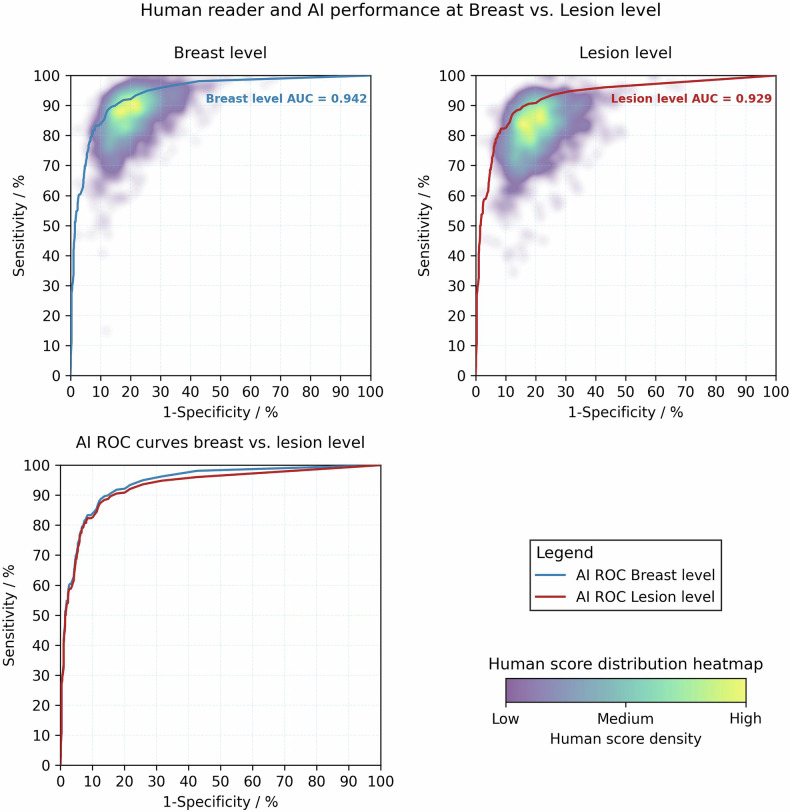
Receiver operating curve (ROC) illustrating diagnostic performance of AI at breast and lesion level. The AI achieved a significantly greater area under the receiver operating curve (AUC) when making a binary recall decision per breast (top left graph), compared to making the recall decision and having to localise the malignant lesion (top right graph), AUC difference −0.013, *p* < 0.01. The heatmaps show the distribution of human readers’ average sensitivity and specificity scores. High-density areas (yellow) indicate where human readers most frequently scored. Each human is represented once by their mean sensitivity/specificity pairs for the schemes they have read

The mean AUC across all ten schemes for humans was 0.878 (standard deviation (SD) 0.029) at breast level, and 0.851 (SD 0.037) at lesion level, resulting in an AUC difference of −0.027, *p* < 0.01, Table 2. The mean AUC of AI was 0.942 at breast level. At the lesion level this decreased to 0.929, AUC difference −0.013, *p* < 0.01, Fig. 3.

**Table 2:** The diagnostic performance (area under the receiver operating curve, AUC) of humans and AI reading the 600 PERFORMS cases included in the study at the breast and lesion level

Comparison	Breast Level	Lesion Level	AUC difference	*z*	*p*
Human mean AUC (SD)	0.878 (0.029)	0.851 (0.037)	0.027	117.153	< 0.01
AI AUC (95% CI)	0.942 (0.927, 0.956)	0.929 (0.911, 0.946)	0.013	2.871	< 0.01

The *p*-value compares the performance of humans and AI at the breast and lesion level using a *z*-test score (*z*)

*SD* standard deviation of human performance across the ten schemes, *95% CI* 95% confidence intervals

Using Stouffer’s method to combine *z*-scores from the ten test schemes, overall human AUC performance was inferior to that of AI at breast level (*z* = 2.983, *p* < 0.01). Using the same method at the lesion level, a similar pattern was demonstrated, with overall human AUC inferior to that of AI (*z* = 3.379, *p* < 0.01).

### Comparison of sensitivity for humans and AI at the breast and lesion level

#### Developer-recommended threshold

At breast level the sensitivity of the average human reader was 87.5% (278/318). No evidence of a difference was found when this was compared to the sensitivity of the AI at the developer-recommended threshold, 88.7% (282/318), *p* = 0.62, Table 3. At lesion level no difference was observed between the average human reader and AI, OR 0.71 (95% CI 0.46, 1.10), *p* = 0.12.

**Table 3:** Comparison of the sensitivity of the average human reader and AI at the breast and lesion level

			AI recall threshold scores			
Metric	Average human reader		Developer recommended recall threshold		Recall threshold matching human specificity	
	Breast	Lesion	Breast level *(T:* ≥ *10)*	Lesion level *(T:* ≥ *10)*	Breast level *(T:* ≥ *4.5)*	Lesion level *(T:* ≥ *4.5)*
True positive findings	278	273	282	287	293	298
False negative findings	40	55	36	41	25	30
Sensitivity (%)	**87.5**	**83.2**	**88.7**	**87.5**	**92.1**	**90.9**
Odds ratio (95% CI)	-	-	0.89 (0.55, 1.43)	0.71 (0.46, 1.10)	0.59 (0.35, 1.00)	0.50 (0.31, 0.80)
*p*-value	-	-	0.62	0.12	0.051	< 0.01

The AI thresholds were calibrated to match the developer’s recommended threshold of ≥ 10, and at a threshold where human specificity was matched (≥ 4.5), enabling a direct comparison of sensitivity

*T* AI threshold for recall, *95% CI* 95% confidence intervals

*p*-values were calculated compared to AI performance to the average “modelled” human reader using odds ratios as described by Kirkwood and Sterne (15)

#### When matching human specificity

At a recall threshold of ≥ 4.5 AI matches, the average human specificity at breast level. The average human recalled 278 breasts but failed to recall 40, meanwhile AI at a threshold of ≥ 4.5 recalled 293 breasts but failed to recall 25, giving sensitivities of 87.5% and 92.1%, respectively (*p* = 0.051). At the lesion level, the average human detected and correctly recalled 273 lesions but failed to recall 55 lesions, AI meanwhile detected and correctly recalled 298 lesions but did not recall 30 lesions, giving sensitivities of 83.2% and 90.9%, respectively (*p* < 0.01).

### Comparison of specificity for humans and AI at the breast level

#### Developer-recommended threshold

The specificity of the average human reader was 79.2% (698/882). This was significantly inferior to the AI specificity of 87.4% (771/882), OR 0.55, *p* < 0.01, Table 4.

**Table 4:** Comparison of the specificity of the average human reader and AI at the breast level

		AI recall threshold scores	
Metric	Average human reader	Developer recommended recall threshold (*T*: ≥ 10)	Recall threshold matching human breast level sensitivity (*T*: ≥ 10.5)
True negative findings	698	771	775
False positive findings	184	111	107
Specificity (%)	**79.2**	**87.4**	**87.9**
Odds ratio (95% CI)	-	0.55 (0.42, 0.71)	0.52 (0.40, 0.68)
*p*-value	-	< 0 .001	< 0 .001

The AI thresholds were calibrated to match the developer’s recommended threshold of *≥ 10*, and at a threshold where human sensitivity was matched at breast level (*≥ 10.5*), enabling a direct comparison of sensitivity

*T* AI threshold for recall, *95% CI* 95% confidence intervals

*p*-values were calculated compared to AI performance to the average “modelled” human reader using odds ratios as described by Kirkwood and Sterne (15)

#### When matching human sensitivity

The AI threshold for recall, which matched the average human sensitivity at breast level, was ≥ 10.5. When comparing specificity at this threshold, the average human reader recalled 184 breasts incorrectly, compared to AI’s 107. This resulted in an inferior specificity for humans compared to AI when matched for sensitivity, 79.2% vs. 87.9%, respectively, OR 0.52, *p* < 0.01.

### Malignant breasts with discordance between breast and lesion level score for AI

**Fig. 4:**
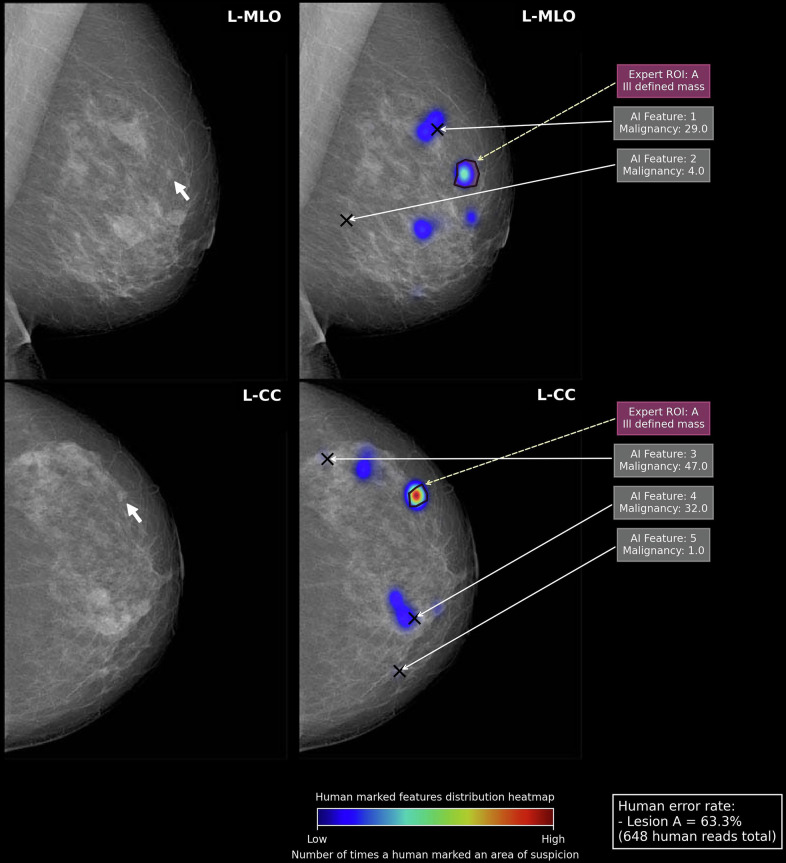
Left panel, A left mediolateral oblique (MLO) and craniocaudal (CC) unadulterated mammogram shows an 8-mm ill-defined mass (white arrowhead) in the upper outer quadrant, which, after biopsy, was determined to be an invasive grade 2 ductal carcinoma. In Fig. 4, right panel, the same ROI is represented by a purple polygon annotated by an expert radiologist (Expert ROI: A). The black crosses symbolise AI regions of interest, along with their respective suspicion of malignancy score. Five AI ROIs were marked, numbered 1 to 5. The heatmaps illustrate how often a human reporting on the case selected a perceived abnormality. A red colour (high density) signifies that a high proportion of humans selected that region, and a blue colour (low density) signifies a smaller number of humans selected that region. The human error rate for the lesion (i.e. the proportion of humans who did not localise and/or assign a score above the threshold for recall out of those who read the case) is also outlined. In the example, the highest score assigned to the breast (for any ROI) was 47 (AI Feature 3, CC view, right panel). Therefore, AI appropriately recalled this case for further investigation at the breast level. Over one third of human readers (36.7%, *n* = 238/648) appropriately localised and recalled the ROI in both views (with more marking it in the CC view). However, AI failed to localise the lesion in question, instead marking an ROI superolateral to this. If the case was reviewed during arbitration, the ability of the human panel to understand the basis of AI’s recall decision is crucial, especially in such cases where there is discordance between the locations suspicious for malignancy between readers. AI ROIs cannot always be assumed to align with the human ROIs. However, this ROI could represent a malignancy not detected or biopsied by human screening readers at the time

**Fig. 5:**
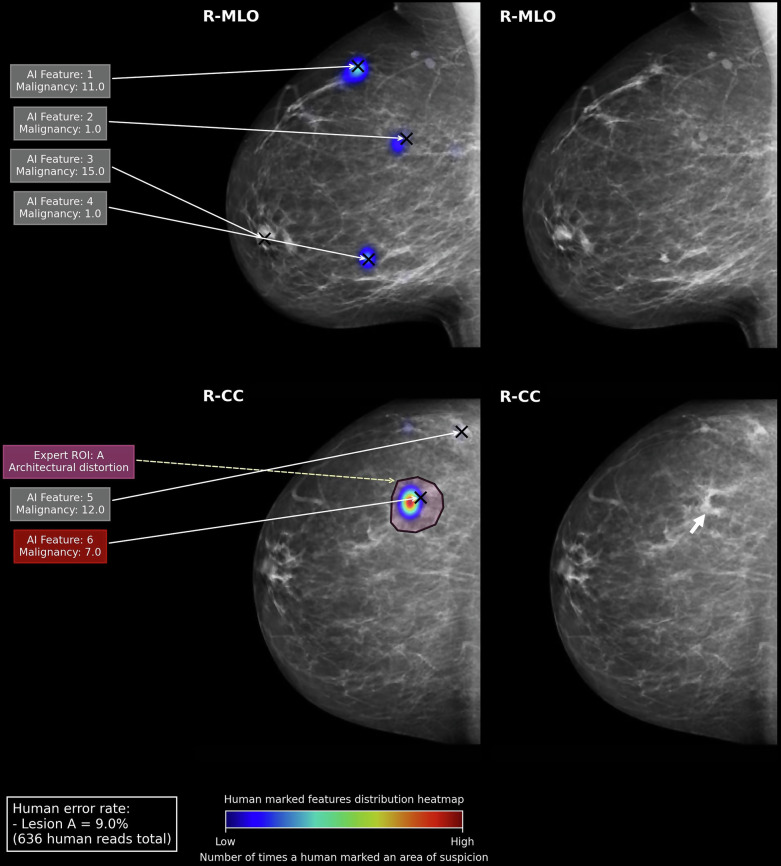
**Right panel:** A 17-mm area of architectural distortion (white arrowhead) is only visible in the right CC view. After biopsy, it was determined to be a histological grade 3 invasive ductal carcinoma. In the **left panel**, the AI correctly localised the malignant ROI (AI Feature 6, score = 7), however, assigns a score lower than the developer’s recommended threshold for recall (≥ 10), suggesting a lower suspicion of malignancy for this particular ROI. Instead, it assigns its highest score (AI Feature 3, score = 15) to an ROI posterior to the nipple. An area in which little or no humans have suggested as an ROI. This would warrant a recall for the breast. If this scenario transpired in real clinical practice, the patient may have been subjected to an unnecessary biopsy due to the AI. The human error rate for the lesion was 9.0% (*n* = 57/636)

**Fig. 6:**
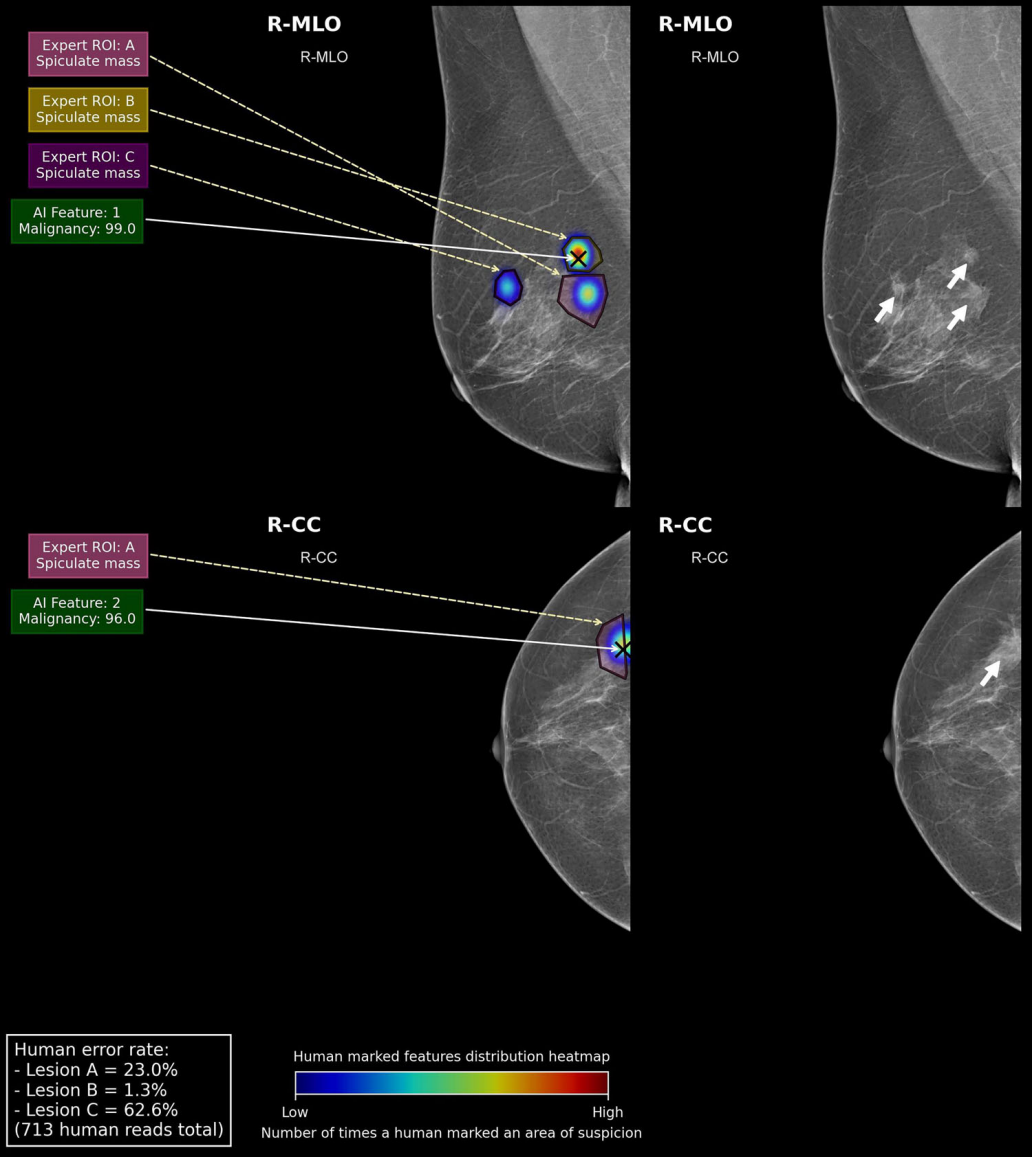
**Right panel:** On the MLO view, there are three spiculate masses demonstrated by the white arrowheads. The posterior mass also contains microcalcifications. The right CC view demonstrates a lesion laterally. The case is highly suspicious of multifocal carcinoma and requires recall for further assessment. Pathology revealed multifocal grade 2 invasive ductal carcinoma. In the **left panel**, AI correctly localises and recalls Expert ROIs A & B, assigning suspicion of malignancy scores of 96 and 99, respectively, to each. However, AI fails to recall the final lesion (Expert ROI: C) as suspicious. The human error rate for this lesion was 62.6% (*n *= 446/713). The inability of AI to recognise this extra lesion, which is located anterior and away from the other two adjacent lesions, may have altered the operative strategy for this patient from a mastectomy for multifocal cancer to breast-conserving surgery

AI assigned discordant scores at the breast and lesion level in a total of five breasts, which contained eight malignant lesions in total. At breast level, AI would have correctly recalled all five breasts with a recall threshold of ≥ 10. However, at the lesion level, five of the eight lesions (62.5%) would not have been recalled, and four of the eight lesions (50%) were not correctly localised by the AI. Examples of discrepant cases are illustrated in Figs. 4, 5 and 6. Out of all malignant lesions in the dataset, AI failed to localise 4%, *n* = 13. The median human error rate for these lesions was 62.6% (quartile 1: 41.8%, quartile 3: 72.8%). They consisted of 3 spiculate masses, 3 ill-defined masses, 2 well-defined masses, 2 areas of calcification, 2 asymmetric densities and 1 area of architectural distortion.

## Discussion

Understanding where AI has marked a region of interest is important for its explainability, and for the follow-up for those who are recalled. However, most studies evaluate if AI correctly recalls a malignant breast and do not consider if AI has correctly localised the malignant lesion in question. We retrospectively compared a single commercial mammography AI algorithm using 1200 breasts in its performance in localising 328 pathologically proven cancers at the breast and lesion level. Overall diagnostic performance for AI was significantly inferior at the lesion level compared to its performance at the breast level (AUC: 0.929 vs. 0.942, respectively, *p* < 0.01). This is reflected in an AI sensitivity of 88.7% (282 of 318 malignant breasts recalled) at breast level vs. 87.5% at lesion level (287 of 238 malignant lesions recalled) at the developer-recommended threshold for recall. Thus, when AI was evaluated in its ability to correctly localise and recall a malignancy, instead of its ability only to classify a breast as cancerous or not, its performance dropped. However, a greater decrease was observed for a similar comparison of the diagnostic performance of 1,258 National Health Service Breast Screening Programme human readers at breast and lesion level (AUC: 0.878 vs. 0.851, respectively, *p* < 0.01). This is reflected by a more pronounced decrease in the average sensitivity from breast (87.5%, 278 of 318 malignant breasts recalled) to lesion (83.2%, 273 of 328 malignant lesions recalled) level amongst humans. On direct comparison to humans, AI’s AUC was superior at the breast and lesion level (*ps* < 0.01, respectively). This is reflected in a superior AI specificity of 87.4% (771 of 882 TN findings), compared to humans, 79.2% (698 of 882 TN findings, *p* < 0.01). A difference was also observed in AI and human sensitivity at the lesion level when it was calibrated to match human specificity (90.9% vs. 83.2%, respectively, *p* < 0.01). Our results suggest that AI’s diagnostic performance during mammography is similar or supersedes that of humans, but variation exists in its image and lesion-level classification of malignancies. Our findings support the notion of implementing AI into a prospective screening workflow, where the localisation of malignancies is beneficial to patients and the screening process.

Several studies have shown that AI can effectively detect interval cancers that were initially missed by human assessment on screening mammograms [19–21]. Due to their negative prognoses for patients, correctly localising them for biopsy is important to any screening programme [22]. As AI is implemented into future screening programmes, it is likely to recall extra cases that contain lesions not visible to the naked human eye. This was observed in a prospective paired reader study in Sweden, where the same AI model used in this study recalled an extra 19 patients who were initially dismissed by two humans. The AI ROIs were subsequently biopsied, and all confirmed malignant [3]. Indeed, in one of the few lesion-level studies, an AI was able to correctly localise 78% (*n* = 93) of cancers that were missed during initial screening and presented as interval cancers in a case-control format. In approximately one quarter (26%, *n* = 24 out of 93) of these cases, the AI was able to correctly localise a malignancy, where, according to a retrospective review of the mammogram by a panel of four specialist breast radiologists, no radiological features of cancer were present [7]. In our study, when the AI threshold for recall was matched to human specificity, the AI recalled an extra 25 cancerous lesions compared to the average human reader. These studies illustrate the importance of assessing AI at the lesion level as opposed to the breast level. AI is likely to recall extra cases [3, 7, 19–21], therefore, we as humans must understand its additional ROIs during the reading, arbitration and follow-up process, in order to optimise the human-AI relationship and to minimise unnecessary investigations or biopsies due to automation bias [23].

As we move towards the installation of AI into prospective studies, we can begin to understand the tangible impact AI may have on treatment outcomes for patients, as opposed to focussing solely on standard diagnostic screening metrics. In a prospective analysis, a commercial AI company was installed as a silent reader in an NHSBSP double human reading workflow. Humans did not have access to the AI suggestions at the time of screening. However, discordant cases between AI and the double reading outcomes were regularly reviewed and were recalled to a dedicated clinic if deemed appropriate. In one case, a female was initially diagnosed with unifocal disease by a human double read and subsequently underwent treatment. However, AI had suggested an additional ROI on the screening mammogram, so the patient was recalled post-operatively and was diagnosed with multifocal disease, thus undergoing a completion mastectomy. The final accurate diagnosis of the patient was only possible through a lesion-level analysis of the breast [2]. If the accuracy of AI lesion level suggestions had been considered from the outset, the additional risk of two operations, instead of one, may have been avoidable. In our study, we focussed on five cases where there was a discrepancy between the AI at the breast and the lesion level in order to identify possible changes in diagnostic or management strategies based on the respective findings. In a similar scenario of multifocal disease (Fig. 6), the AI in our study failed to localise a third and final malignant ROI in the upper breast. Although the breast was correctly recalled by AI, its inability to recognise the extra lesion in this case may have led to a similar sub-optimal operative course for the patient as in the prospective study [2].

Study limitations include its retrospective nature, meaning that although stand-alone AI performance was evaluated, its influence on human reporting is not evaluated during concurrent reading. A further limitation includes the use of test sets enriched with cancer cases. This may contribute to a ‘laboratory effect,’ where humans are aware of the enrichment, leading to an artificially greater recall rate [24, 25]. AI is unlikely to be affected by such enrichment. Finally, both the breast and lesion level analyses did not consider prior images during human or AI interpretation. In real clinical practice, humans are able to identify new ROIs more accurately by comparing prevalent and incident round screening mammograms.

By using challenging mammogram cases used in the routine external quality assurance of NHSBSP human readers, AI showed high levels of diagnostic accuracy, exhibiting a superior specificity and a greater sensitivity to humans at the lesion level. To our knowledge, this constitutes the largest cohort of specialised breast cancer screening readers compared to AI. While AI performance decreased when evaluated at the lesion level compared to the breast level, the decline in human performance for the same comparison was greater. Lesion-level AI analyses are seldom reported in the literature [2, 7, 8], but they could have implications on the human-AI relationship during assisted mammography reading, particularly in cases where there is discordance. An AI tool that can report at the lesion level accurately provides positive insight into its “thought” process, which is particularly important as we move towards the prospective implementation of AI, benefiting the human reporter, arbitration panels and patients alike.

## Supplementary information

ELECTRONIC SUPPLEMENTARY MATERIAL

## Abbreviations

AI: Artificial intelligence
AUC: Area under the curve
CC: Craniocaudal
CI: 95% Confidence intervals
FFDM: Full field digital mammogram
FN: False negative
MLO: Mediolateral oblique
NHSBSP: National Health Service Breast Screening Programme
OR: Odds ratio
PERFORMS: Personal Performance in Mammographic Screening
RCR: Royal College of Radiologists
ROI: Regions of interest
SD: Standard deviation
TN: True negative
TP: True positive
