# Supplementary material for: The evaluation of artificial intelligence in mammography-based breast cancer screening: Is breast-level analysis enough?
Source: Eur Radiol. 2025 Jun 25;35(12):8230–43. doi: 10.1007/s00330-025-11733-8 (PMC12634717; doi:10.1007/s00330-025-11733-8)
Supplement: Supplementary file 1 — ELECTRONIC SUPPLEMENTARY MATERIAL [file 330_2025_11733_MOESM1_ESM.pdf]

The Evaluation of Artificial Intelligence in Mammography Based Breast Cancer Screening: Is Breast-Level Analysis Enough?

Electronic Supplementary Material (ESM)

Eur Radiol (2025) Taib AG, Partridge GJW, Yao L, Darker I, Chen Y et al.

Supplementary materials

Hit detection algorithm

Due to the scale of the human dataset, with 7,015 unique human-scheme reads equating to more than 420,000 individual case reads, performing a lesion-based analysis with accurate lesion localisation necessitated an automated hit detection approach.

All abnormal cases included had region of interest (ROI) polygons drawn around lesions, defined by a series of x, y image coordinates that make up each vertex. When humans or AI marked a feature or ROI on an image in PERFORMS, they marked a single point defined by a singular x, y image coordinate. Therefore, programmatically, it was possible to determine whether a human/AI marked feature was marked within the boundary defined by the ground truth ROI polygon (classified as a 'hit' [Figure S1]) or if the human/AI feature was marked outside of the ground truth ROI polygon (classified as a 'miss' [Figure S2 and S3]). For 'misses', the distance from the human/AI feature to the nearest edge of the ground truth polygon was calculated, and another metric was calculated using this distance and accounting for the area (size) of the ROI polygon. This distance, and its cognate metric, were then used to reclassify 'near misses' as 'hits' (Figure S3 and S4).

The above automated method was validated against a sample of manually collected data, where three members of the team manually recorded 'hits' and 'misses' for all AI case reads.

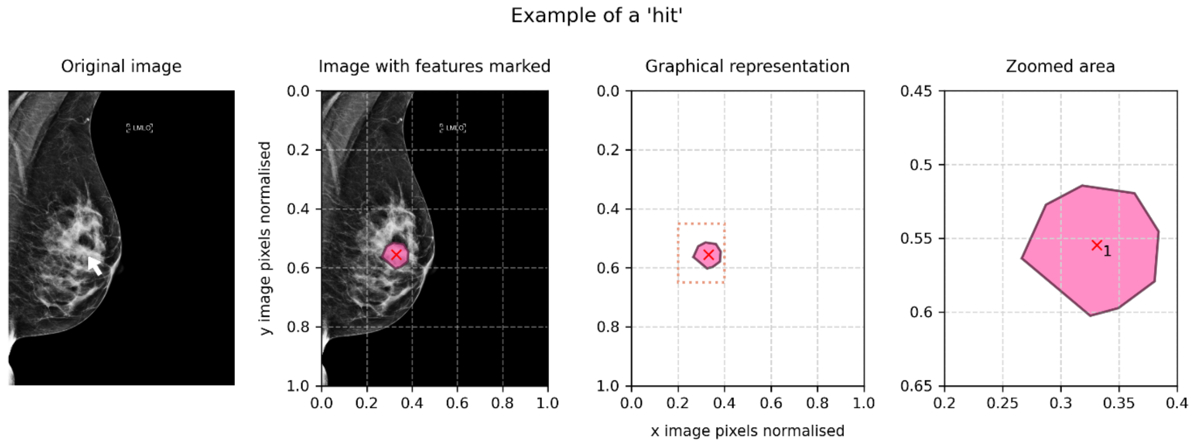

Figure S1. Example of a 'hit'. The original image (left) shows a left mediolateral oblique (MLO) projection with a spiculate mass lying centrally (arrow). This image is then shown with the graphical overlay (normalised pixel coordinates), with the expert drawn ROI denoted by the pink polygon, and a user feature denoted by the red 'x'. This is then translated to a pure graphical representation, with the ROI and user feature zoomed (right).

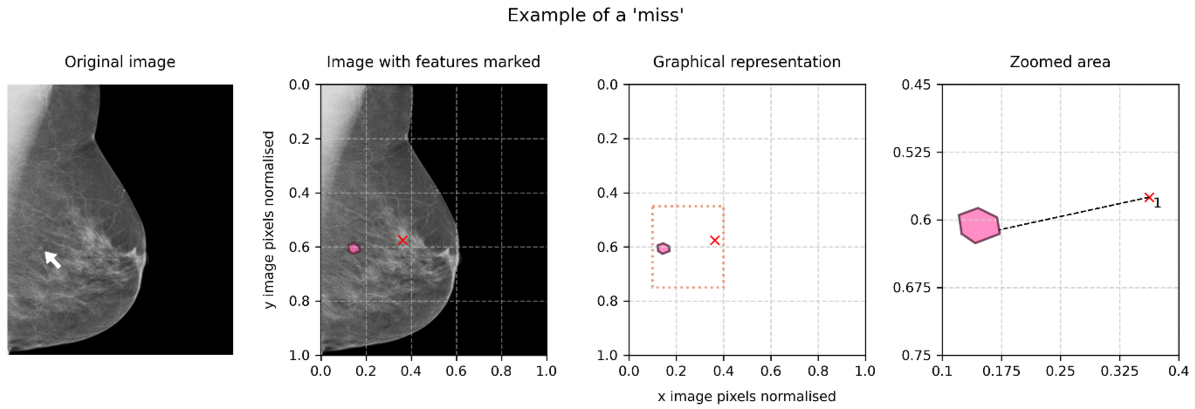

Figure S2. Example of a ‘miss’. The original image (left) shows an MLO view of the left breast with a small ill-defined mass denoted by the arrow. The image is also shown with the graphical overlay, with the expert drawn ROI denoted by the pink polygon, and a user feature denoted by the red ‘x’. This is translated to a pure graphical representation, with the ROI and user feature zoomed (right). As seen in the mammographic image, the user feature is clearly marked anterior from the ROI, in the incorrect region. Indeed, the distance denoted by the black dashed line between the ROI and user feature in the zoomed plot (right) is greater than the threshold required for ‘near miss’ reclassification. Additionally, the cognate distance-ROI area metric was also greater than the threshold required for ‘near miss’ reclassification.

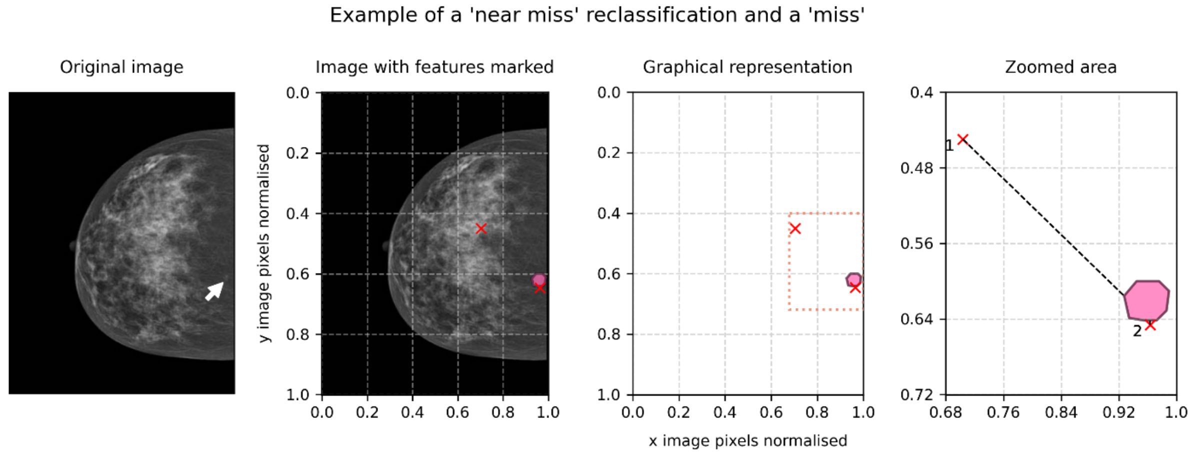

Figure S3. Example of a ‘miss’ and a ‘near miss’ reclassification. The original image (left) shows a right craniocaudal (CC) image with a small spiculated mass along the posterior margin of the breast medially (arrow). In the graphical representations, two user features are marked (denoted 1 and 2 in the zoomed plot [right]). Feature 1 is classified as a ‘miss’, whereas feature 2 is reclassified as a ‘hit’, since the distance between the feature and the ROI is less than the threshold.

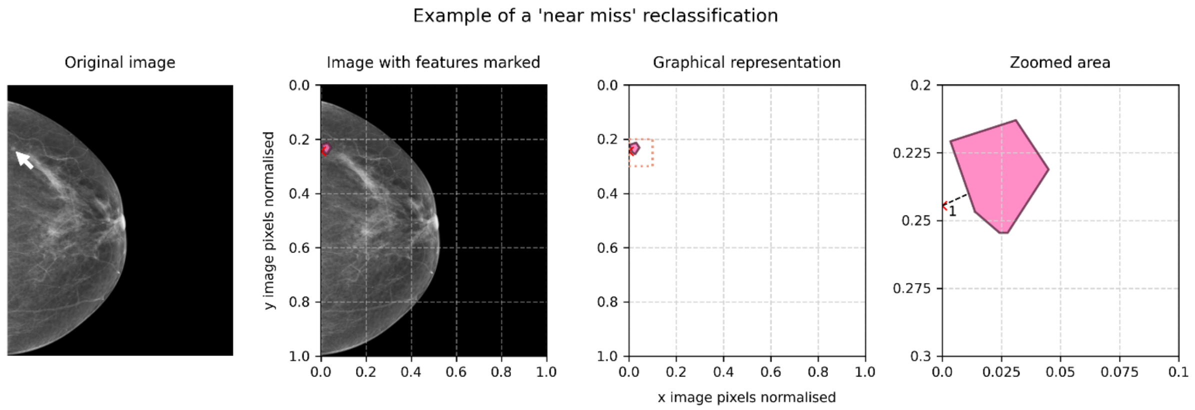

Figure S4. Example of a 'near miss' reclassification. The original image (left) shows a left CC image with a small ill-defined mass marked by the arrow. In this example, the feature marked by the user 'misses' the ROI, and the distance between the feature and the ROI is greater than the distance threshold, so is still classed as a 'miss'. However, the cognate distance-ROI area metric threshold is satisfied, and therefore, the feature is reclassified as a 'hit'.
